# Neoadjuvant apatinib plus S-1 in locally advanced pulmonary adenocarcinoma: A case report and review of the literature

**DOI:** 10.1097/MD.0000000000018767

**Published:** 2020-01-17

**Authors:** Chu Zhang, Xiang Wang, Miao Zhang, Dong Liu, Dun-Peng Yang

**Affiliations:** a Department of Thoracic Surgery, Shaoxing People's Hospital (Shaoxing Hospital, Zhejiang University School of Medicine), Shaoxing; b Department of Thoracic Surgery, Xuzhou Central Hospital Affiliated to Southeast University, Xuzhou, China.

**Keywords:** apatinib, neoadjuvant therapy, pulmonary adenocarcinoma, S-1, vascular endothelial growth factor receptor (VEGFR)

## Abstract

**Rationale:**

About one-third of the lung tumors are staged as locally advanced at the time of initial diagnosis; however, the optimal induction treatment before curative resection has not been elucidated. To date, the evidence regarding the preoperative apatinib plus S-1 for locally advanced pulmonary adenocarcinoma is scarce.

**Patient concerns:**

A 29-year-old female was admitted because of persistent cough, sputum, and chest distress for 2 months.

**Diagnoses:**

Primary pulmonary adenocarcinoma (cT3N2M0, IIIB) with unknown driver gene mutation status.

**Interventions:**

The patient had received 4 months of neoadjuvant therapy using oral apatinib (425 mg daily) plus S-1 (60 mg, twice daily for 4 weeks with a 2-week drug-free interval), followed by anatomical lobectomy with curative intent. Adjuvant apatinib (425 mg daily for a month, and 250 mg daily for another month) plus S-1 at the same dosage were administered for 2 months. Thereafter, maintenance of low-dose S-1 monotherapy (40 mg, twice daily for 4 weeks with a 2-week drug-free interval) was continued for 6 months.

**Outcomes:**

The adverse events were tolerable and well-controlled. A postoperative recurrence-free survival for 2 years and a half up to now was indicated.

**Lessons:**

Preoperative apatinib plus S-1 showed efficacy in locally advanced pulmonary adenocarcinoma. However, high-quality trials are warranted before the recommendation of this therapeutic regimen.

## 1 Introduction

Lung cancer is the most commonly diagnosed cancer (11.6% of the total cases) and the leading cause of cancer death (18.4% of the total cancer deaths).^[1]^ The optimal management including neoadjuvant and adjuvant therapy for stage IIIA/N2 nonsmall cell lung cancer (NSCLC) is yet to be elucidated in the era of targeted therapy and immunotherapy. A network meta-analysis shows that neoadjuvant chemotherapy followed by surgery and adjuvant chemotherapy or radiotherapy has the greatest possibility to be the optimal regimen with the best overall survival and fewest treatment-related deaths for stage IIIA-N2 NSCLC.^[2]^

Apatinib, an oral tyrosine kinase inhibitor targeting vascular endothelial growth factor receptor-2, is effective for a broad range of solid tumors. S-1, an oral anticancer fluoropyrimidine derivative, is active and well tolerated as monotherapy for previously treated, advanced (clinical stage IIIB-IV) or relapsed NSCLC.^[3,4]^ S-1 monotherapy has demonstrated marked activity against NSCLC as well as gastric, colorectal, breast, cervical, and pancreatic cancers.^[5]^ First-line S-1, carboplatin, and antiangiogenetic bevacizumab followed by maintenance S-1 and bevacizumab had been reported to be active in advanced nonsquamous NSCLC.^[6]^ On the contrary, another trial revealed that the addition of bevacizumab to S-1 was not beneficial for patients with previously treated nonsquamous NSCLC.^[7]^ Therefore, it is important to clarify the most suitable agents for use with S-1 and the optimal timing of targeted therapy for lung cancer.

To the best of our knowledge, the available evidence regarding the application of apatinib plus S-1 for locally advanced pulmonary adenocarcinoma is rare. We herein presented a case of locally advanced pulmonary adenocarcinoma in which partial response was indicated after oral apatinib plus S-1 as induction therapy.

## 2 Case presentation

**Figure 1:**
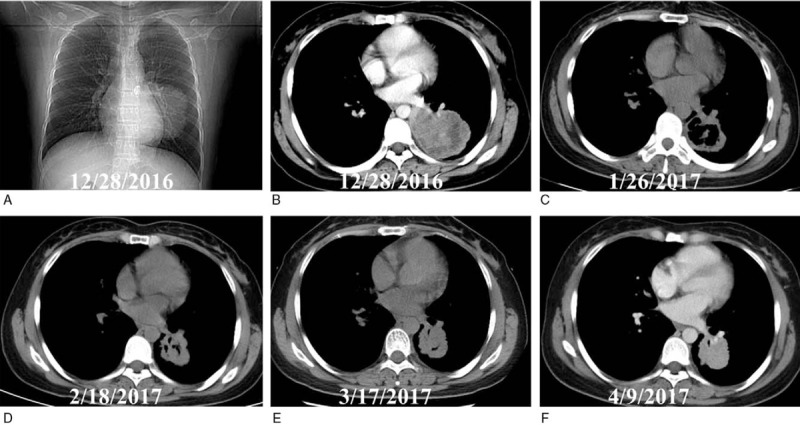
Chest x-ray and CT images of the pulmonary tumor during the induction treatment. A, X-ray on admission showed a bulky mass located in the left lower lobe. B, CT showed that the mass was 70 mm × 60 mm in size. C, One month after oral apatinib plus S-1, the tumor indicated partial remission (PR) measuring 43 mm × 54 mm with a necrotic cavity. D, Two months after induction therapy, the tumor showed stable disease (SD) measuring 44 mm × 37 mm. E, The lesion was 41 mm × 40 mm in size 3 months after treatment and SD was indicated. F, Four months after, the tumor was slightly enlarged measuring about 41 mm × 42 mm before surgery. CT = computed tomography.

In December 2016, a 29-year-old female nonsmoker was admitted for persistent cough, sputum, and chest distress for 2 months, without hemoptysis, hoarseness, chest pain, or significant loss of body weight. Her previous medical history was unremarkable. The Eastern Cooperative Oncology Group (ECOG) performance status was 0. Chest x-ray on admission revealed a mass in left lower lobe (Fig. 1A). In addition, laboratory tests showed elevated serum carcinoembryonic antigen (CEA), neuron-specific enolase (NSE), and cytokeratin-19 fragment (CYFRA 21-1). Further computed tomography (CT) indicated an irregular tumor measuring 70 mm × 60 mm in size (Fig. 1B) and enlarged mediastinal lymph nodes.

Bronchoscopic biopsy and pathological stain revealed the diagnosis of primary pulmonary adenocarcinoma. Distal metastasis was excluded by contrast-enhanced abdomen CT, cranial magnetic resonance, and whole-body bone emission CT. Then this case was staged as cT3N2M0, IIIB according to the 8th edition of TNM staging system for lung cancer.^[8,9]^

Meanwhile, the patient refused hospitalization and the standard first-line intravenous pemetrexed and carboplatin for personal reasons. However, genetic testing for the mutation status of epidermal growth factor receptor (EGFR), anaplastic lymphoma kinase, and programmed cell death protein 1 was not performed because it was not covered by her health insurance. Thus, EGFR-targeted agents or immunotherapy were not considered as the first therapeutic option. After a multidisciplinary evaluation, oral apatinib (425 mg daily) plus S-1 (120 mg per day for 4-week and 2-week withdrawal as her body surface area was > 1.5 m^2^) was administered. During the induction therapy in outpatient clinic, CT and laboratory tests for serum CEA, NSE, and CYFRA21-1 were conducted regularly for efficacy evaluation according to Response Evaluation Criteria in Solid Tumors (RECIST 1.1), and the adverse events (AEs) were recorded in accordance with the National Cancer Institute Common Terminology Criteria for Adverse Events version 4.0.

**Figure 2:**
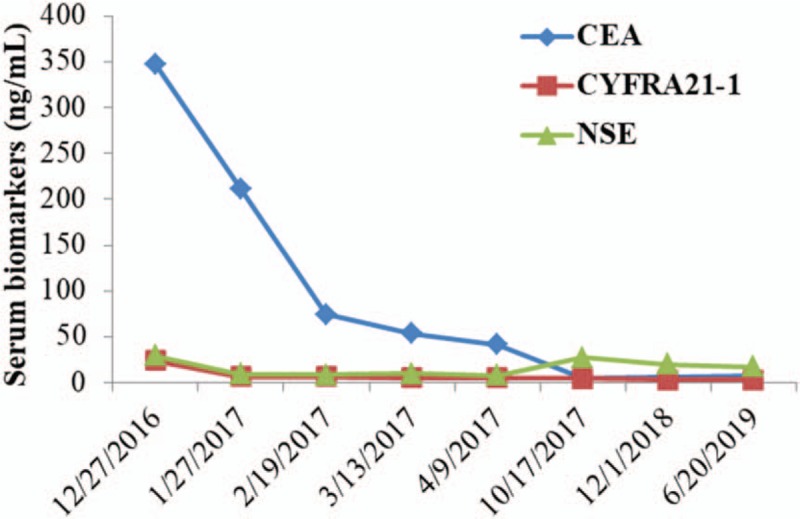
Changes of serum CEA, NSE, and CYFRA21-1 levels during the treatment. CEA = carcinoembryonic antigen, CYFRA 21-1 = cytokeratin-19 fragment, NSE = neuron-specific enolase.

Encouragingly, the pulmonary adenocarcinoma indicated partial remission after 1 month of preoperative apatinib plus S-1 and stable disease during the next 3 months of the medical treatment (Fig. 1). Similarly, the serum CEA, NSE, and CYFRA21-1 were decreased steadily (Fig. 2). Grade 3 anemia, anorexia, hand-foot syndrome, and oral mucositis were observed and controlled effectively. No grade 4 toxicities were recorded during the neoadjuvant therapy.

On April 9, 2017, the pulmonary adenocarcinoma was slightly enlarged (Fig. 1F) but it was considered to be resectable. Therefore, salvage lobectomy using fast-track protocol was scheduled as her ECOG score was 0. Anatomical lobectomy and mediastinal lymph node dissection was performed on April 13, 2017. Pulmonary vein-first approach was utilized during the surgery, with the aim of diminishing the risk of intraoperative tumor dissemination.^[10]^ Prophylactic ligation of the thoracic duct was not performed. A 26 French tube was used for chest drainage. The operating time was 75 minutes, with estimated blood loss of nearly 100 mL.

Ultrasound-guided serratus anterior plane block was applied for pain relief. Her postoperative course was mainly uneventful, and she was discharged 5 days after surgery. R0 resection was achieved, and the maximal diameter of the tumor in specimen was 60 mm × 35 mm. The pathological diagnosis was poorly differentiated lung adenocarcinoma with visceral pleura invasion (pT3N0M0, IIB).

Three weeks after the operation, adjuvant apatinib (425 mg daily for a month, and 250 mg daily for another month) plus S-1 (120 mg daily for 4 and 2-week withdrawal) were administered for 2 months, and then the apatinib was discontinued due to grade 3 hand-foot syndrome and grade 4 elevated serum aspartate aminotransferase and alanine aminotransferase. Thereafter, low-dose S-1 (40 mg, twice daily at the same schedule) as maintenance therapy was continued for another 6 months, although local recurrence or distant metastasis of pulmonary adenocarcinoma was not observed during the follow-up. The serum biomarkers of CEA, NSE, and CYFRA 21-1 were also in normal range (Fig. 2). This patient demonstrated a PFS of 2 and a half years up to November 2019.

## 3 Discussion

To the best of our knowledge, this is the first case report of first-line induction apatinib plus S-1 for locally advanced pulmonary adenocarcinoma, and this treatment regimen showed a promising effect on survival of the patient.

It is reported that one-third of the NSCLC patients are found to have locally advanced tumors at the time of initial diagnosis,^[11]^ and neoadjuvant therapy followed by surgery and adjuvant therapy might be the optimal treatment.^[2]^ Down-staging of primary tumor and/or mediastinal lymph nodal metastases after induction therapy are positive prognostic factors in selected patients.^[12]^ Another study indicates that there is a nonsignificant difference between the outcomes of neoadjuvant and adjuvant chemotherapy for IIIA NSCLC patients.^[13]^ Neoadjuvant tyrosine kinase inhibitor (TKI) erlotinib is well tolerated and might improve the resection rate of stage IIIA-N2 EGFR mutation-positive NSCLC, and the next-generation sequencing could be utilized to predict outcomes in these patients.^[14]^ Moreover, large neoadjuvant trials of immunotherapies and targeted therapies in advanced disease are underway.^[15]^

The optimal adjuvant therapy for clinical N2 NSCLC patients who undergo neoadjuvant chemotherapy/immunotherapy and surgery has not been elucidated.^[16]^ Brandt et al^[17]^ report that neoadjuvant or adjuvant chemotherapy is not associated with an improvement in overall survival or PFS among patients with cT2∼4N0∼1M0 NSCLC after radical surgery. Another population-based study shows that patients with cN2 disease but postchemotherapy surgical nodal staging ypN0∼1 and/or lymph node ratio (LNR, which is defined as number of lymph nodes involved by tumor divided by total number of dissected nodes) < 15% do not benefit from adjuvant therapy, whereas the patients with persistent N2 disease and LNR > 15% who receive adjuvant chemoradiotherapy have improved survival,^[18]^ which indicates that aggressive therapy is beneficial to the patients with persistent or high nodal burden disease.

S-1 plus cisplatin in combination with radiotherapy results similar efficacy but better hematological tolerability (lower risk of leukocytopenia and neutropenia) as compared with standard concurrent chemoradiation regimens in locally advanced NSCLC.^[19]^ In addition, S-1 as a third- or fourth-line therapy for wild-type EGFR NSCLC demonstrates numerically better clinical outcomes than erlotinib.^[20]^ Furthermore, postoperative S-1 for 1 year seems feasible for stage IB-IIIA lung cancer with low incidence of AEs.^[21]^ However, there is no consensus regarding the benefit of S-1 maintenance therapy for squamous cell lung cancer.

Apatinib has shown survival benefit in NSCLC trials with a favorable AEs profile.^[22]^ The previously reported studies of S-1 plus TKIs or bevacizumab for lung cancer are listed in Table 1.^[6,7,23–31]^ First-line S-1, carboplatin, and bevacizumab followed by maintenance S-1 and bevacizumab are active for advanced nonsquamous NSCLC.^[6]^ S-1 plus bevacizumab produces modest survival efficacy in second-line treatment for advanced nonsquamous NSCLC.^[25]^ First-line S-1 plus cisplatin with bevacizumab, and pemetrexed plus cisplatin with bevacizumab have similar activity and tolerability in patients with advanced nonsquamous NSCLC.^[29]^ Nevertheless, other trials show that S-1 plus bevacizumab does not provide any additional benefit in terms of PFS for nonsquamous NSCLC patients after failure of platinum-based chemotherapy.^[26,27]^

**Table 1:**
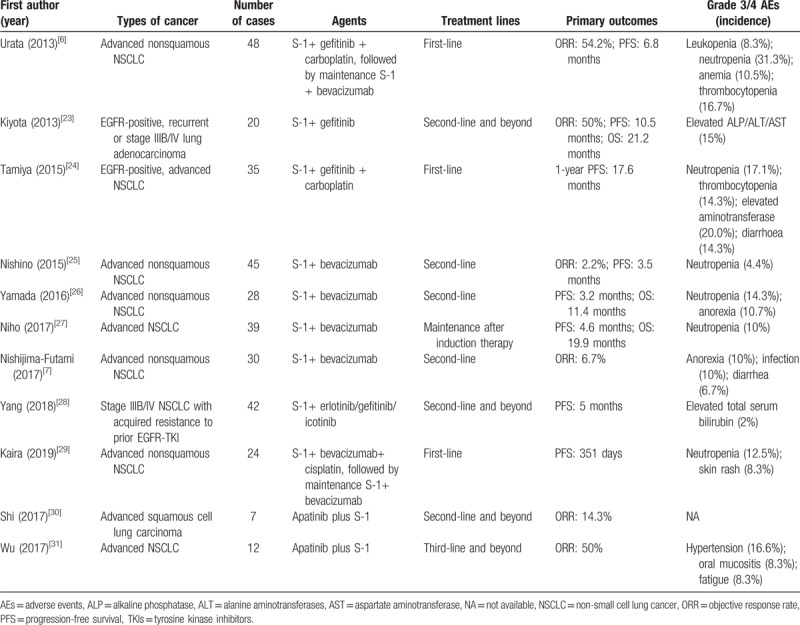
The reported clinical trials evaluating the efficacy of S-1 plus bevacizumab or TKI for lung cancer.

As for the researches regarding S-1 plus TKIs, a phase II trial shows that first-line concurrent carboplatin, S-1, and gefitinib is efficacious in advanced EGFR mutation-positive NSCLC patients.^[24]^ S-1 plus EGFR-TKIs shows synergistic efficacy in stage IIIB-IV NSCLC patients who have experienced prior EGFR-TKI failure because of acquired resistance.^[28]^ Another trial indicates that S-1 plus gefitinib is effective in EGFR mutation-positive pulmonary adenocarcinoma.^[23]^

Based on available reported studies and the presented case, targeted therapy in combination with S-1 might be an alternative option for locally advanced NSCLC. However, well-designed trials for convincing evidence are warranted before the implementation of TKIs or antiangiogenesis agents plus S-1 into therapeutic guideline. The registered trials evaluating the efficacy of TKIs such as gefitinib, anlotinib, and antiangiogenetic agents including bevacizumab and apatinib plus S-1 for lung cancer are summarized in Table 2.

**Table 2:**
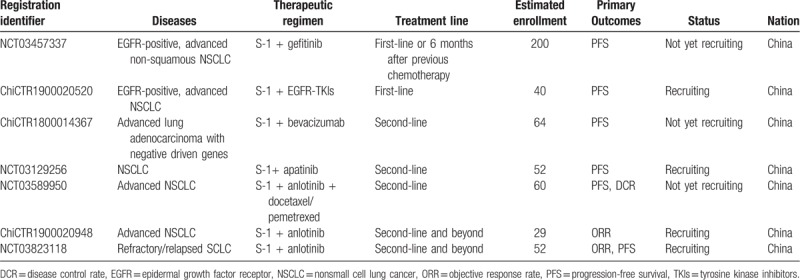
The registered trials evaluating the efficacy of targeted agents plus S-1 for lung cancer.

From this case, there are several questions arise: Is there any reliable efficacy indicators of apatinib plus S-1 for patient selection? How to determine the optimal duration of induction therapy using antiangiogenetic agents? Is adjuvant apatinib plus S-1 necessary for pulmonary adenocarcinoma after R0 resection with ypN0 status (how to avoid over-treatment)? Similarly, did this patient really benefit from the 6-month maintenance therapy using S-1? The role of targeted agents needs to be validated in the era of immunotherapy.

In summary, apatinib plus S-1 showed efficacy in locally advanced pulmonary adenocarcinoma. However, high-quality evidence is needed.

## Author contributions

**Conceptualization:** Chu Zhang, Dong Liu.

**Data curation:** Dong Liu.

**Formal analysis:** Chu Zhang, Miao Zhang.

**Funding acquisition:** Chu Zhang, Xiang Wang.

**Methodology:** Xiang Wang, Miao Zhang, Dun-Peng Yang.

**Supervision:** Dun-Peng Yang.

**Validation:** Miao Zhang, Dun-Peng Yang.

**Writing – original draft:** Chu Zhang, Dong Liu.

**Writing – review & editing:** Xiang Wang, Dong Liu.

Dong Liu orcid: 0000-0003-2071-4548.
